# Supplementary figures and images for: Comparative transcriptomics reveal the common anteroposterior molecular blueprint of adult bilaterian guts
Source: PLoS Biol. 2026 Jan 5;24(1):e3003571. doi: 10.1371/journal.pbio.3003571 (PMC12768270; doi:10.1371/journal.pbio.3003571)

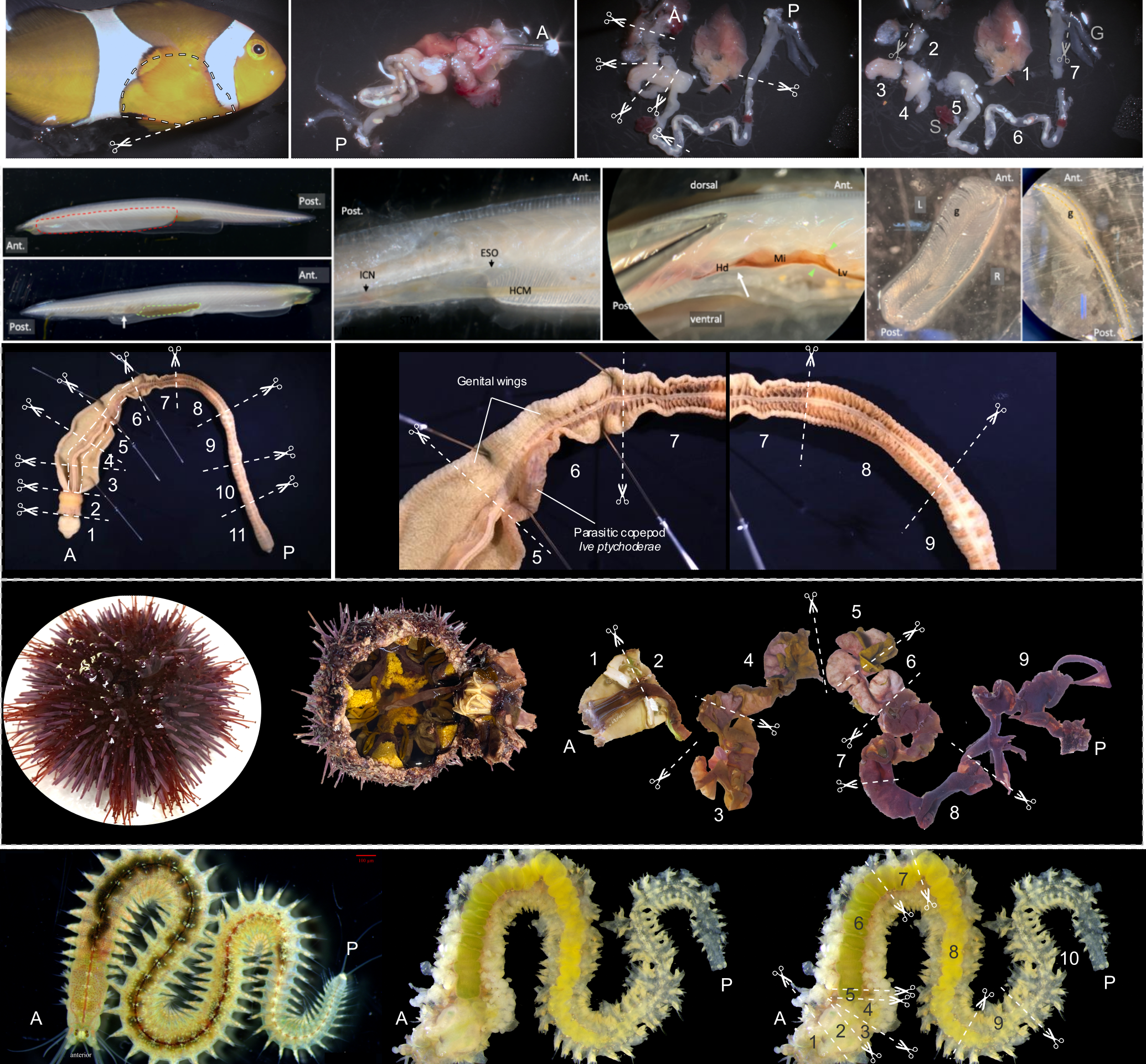

Supplement: S1 Fig — For each species, representative pictures of the dissection process and subsectioning criteria to obtain the final gut segments as illustrated in Fig 1. A, anterior; P, posterior. Dashed lines with scissors, dissection boundary. Segments are numbered according to AP position, matching labels in Fig 1. First row: clownfish gut dissection. Second row: amphioxus gut dissection. Third row: acorn worm sections. Fourth row: sea urchin gut dissection. Fifth row: Platynereis gut dissection. (PNG) [file pbio.3003571.s001.png]

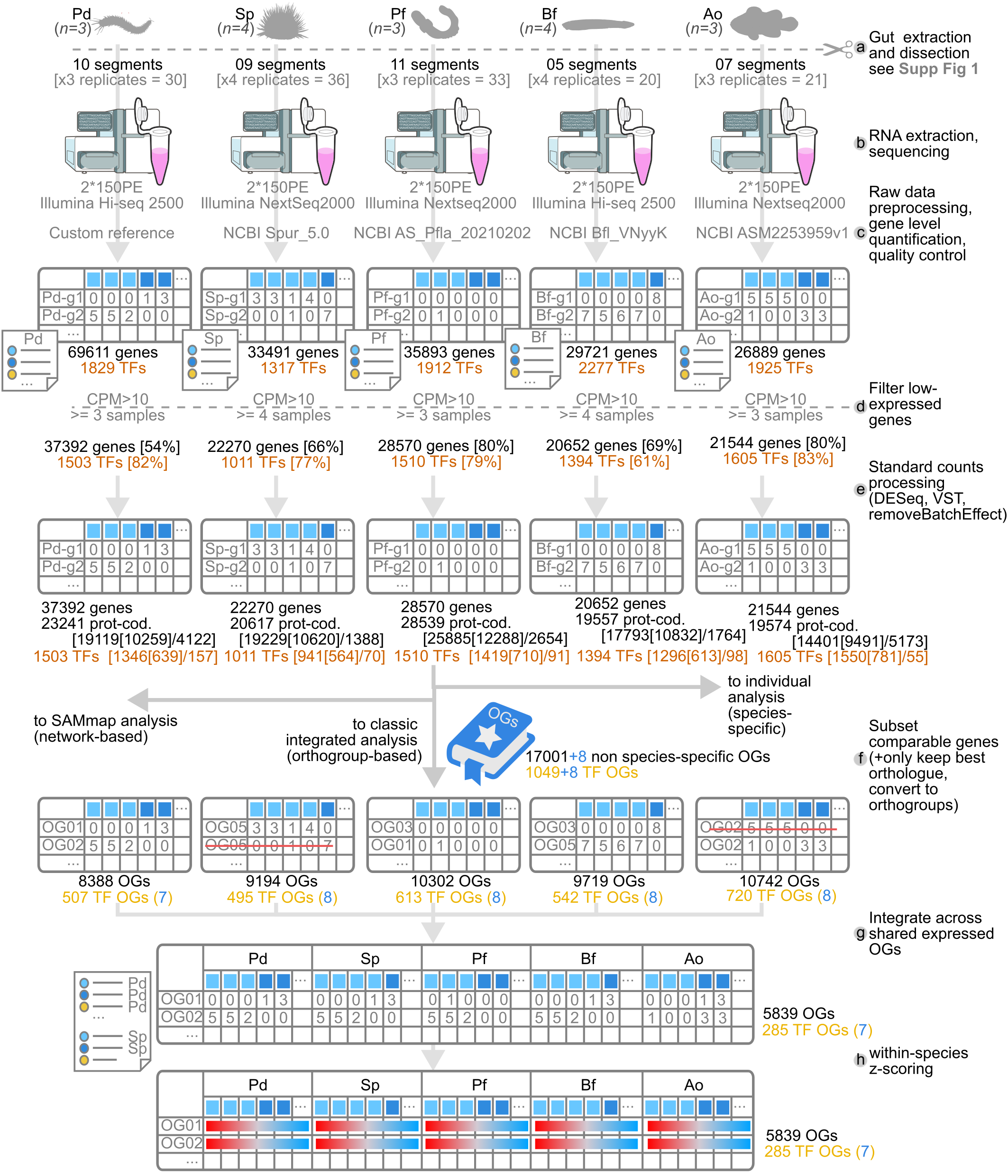

Supplement: S2 Fig — (PNG) [file pbio.3003571.s002.png]

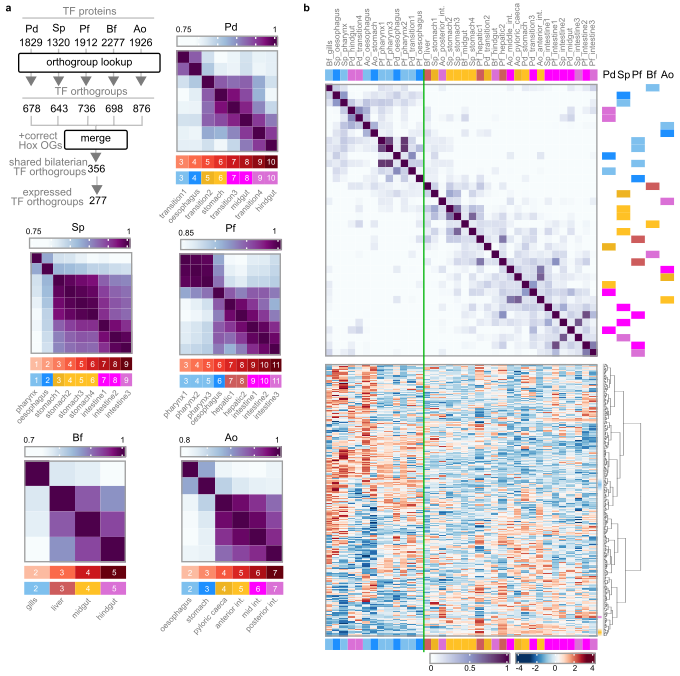

Supplement: S3 Fig — A) Summary of the steps deployed to derive the final set of conserved, gut-expressed transcription factors (TFs) used for cross-species comparison. For each species, heatmap of the Spearman’s Rank Correlation coefficients between segments, based on the expression of these same TFs. Segments are arranged according to R2E seriation, which matches AP position. B) Partial Generalized Association Plot summarizing TF expression data across bilaterian gut segments. Top: heatmap of the Spearman’s Rank Correlation coefficients between all segments, regardless of species. Segments are ordered according to R2E seriation. Bottom: heatmap showing the expression pattern (z-scores) of all TFs considered. TFs (rows) are ordered according to R2E seriation-guided hierarchical clustering. Segments are color-coded by approximate equivalent AP position, as indicated in A. Solid green line indicates qualitative grouping of gut segments based on correlation patterns and known anatomical position. The data underlying this Figure can be found in https://doi.org/10.5281/zenodo.17746910. (PNG) [file pbio.3003571.s003.png]

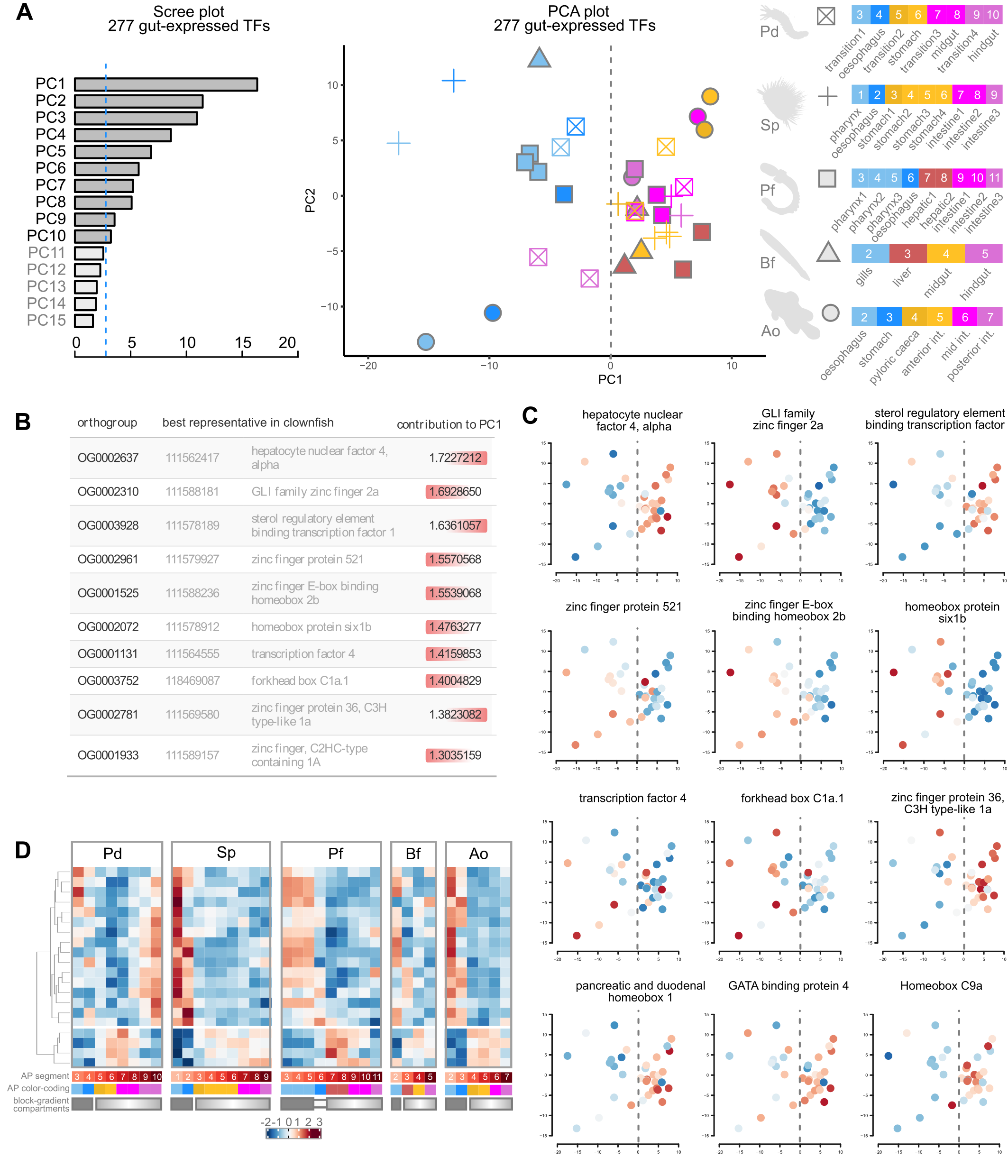

Supplement: S4 Fig — A) Distribution of all gut segments (all species) across the two main Principal Components (PCs; PC1, PC2) and based on the full set of conserved, gut-expressed transcription factors (TFs). Segments are color-coded by approximate equivalent AP position as in the legend provided on the right. Dashed vertical line indicates main separation between clusters. Left: Plot indicating the proportion of variance explained by each PC (scree plot). Blue dashed line: proportion of variance that would be explained if all components had equal contribution. B) Summary table of the top TFs associated with sample separation across PC1. Right/Left red highlight of the contribution scores indicate whether TF expression is driving samples to the right/left of the PC plot, respectively. C) Same data as in the central panel of A, but with segments color-coded by relative level of expression of each of the main PC1 drivers (as in B) and other TFs of interest (bottom row). Red, high; blue, low. Dashed vertical line indicates main separation between clusters. D) For each species, heatmap showing the expression pattern (z-scores) of the main drivers of separation across PC1. TFs (rows) are clustered according to hierarchical clustering. Solid and graded rectangles under the heatmaps indicate segments previously assigned to block and gradient gut compartments, as defined in previous sections. The data underlying this Figure can be found in https://doi.org/10.5281/zenodo.17746910. (PNG) [file pbio.3003571.s004.png]

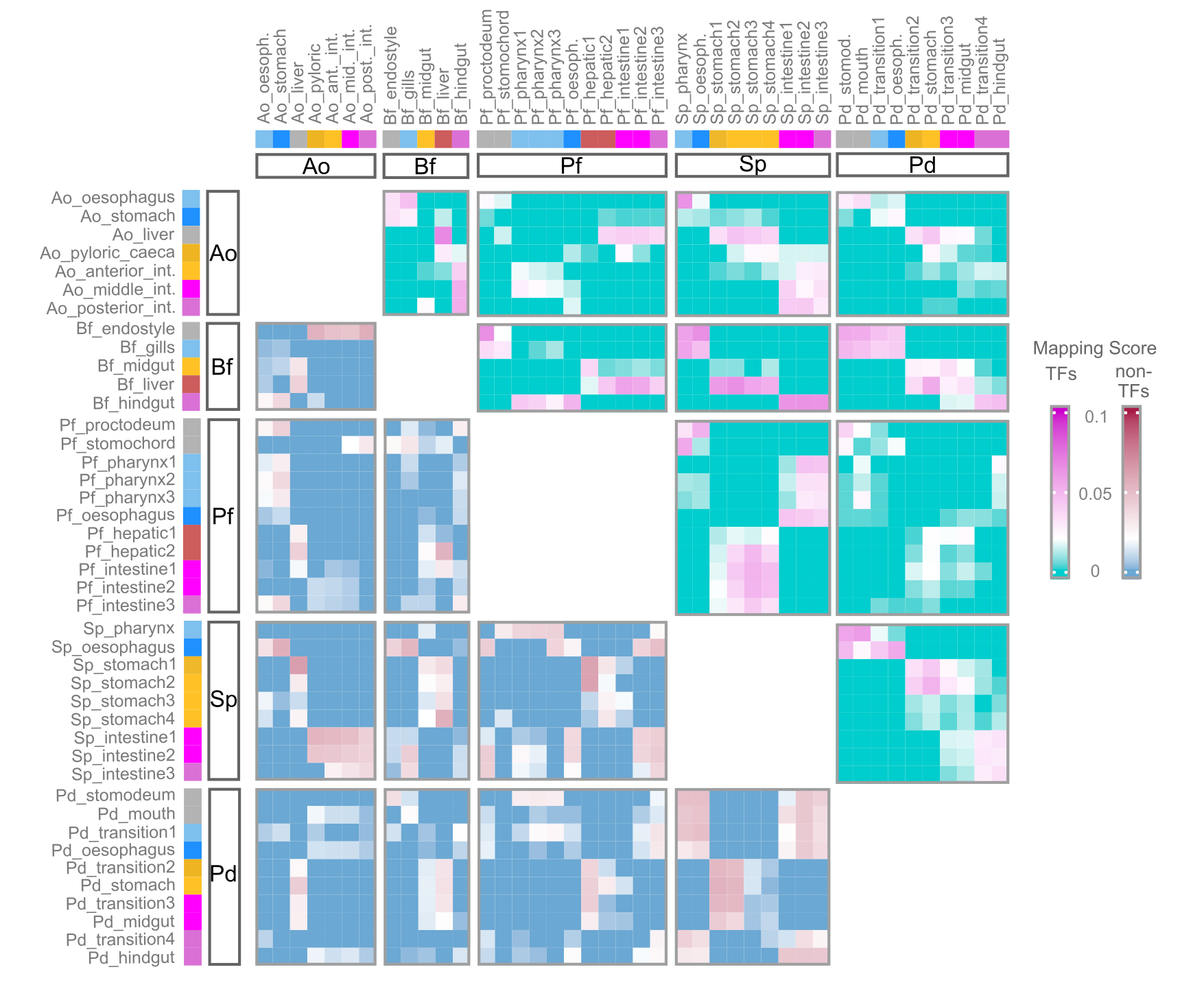

Supplement: S5 Fig — A) Heatmap of SAMap Mapping Scores between gut segments across species, based on the expression of all TF genes (upper triangle, above the diagonal) or all non-TF genes (lower triangle, below the diagonal). In the SAMap approach, all paralogues are included for 1-to-many orthogroups. The data underlying this Figure can be found in https://doi.org/10.5281/zenodo.17746910. (PNG) [file pbio.3003571.s005.png]

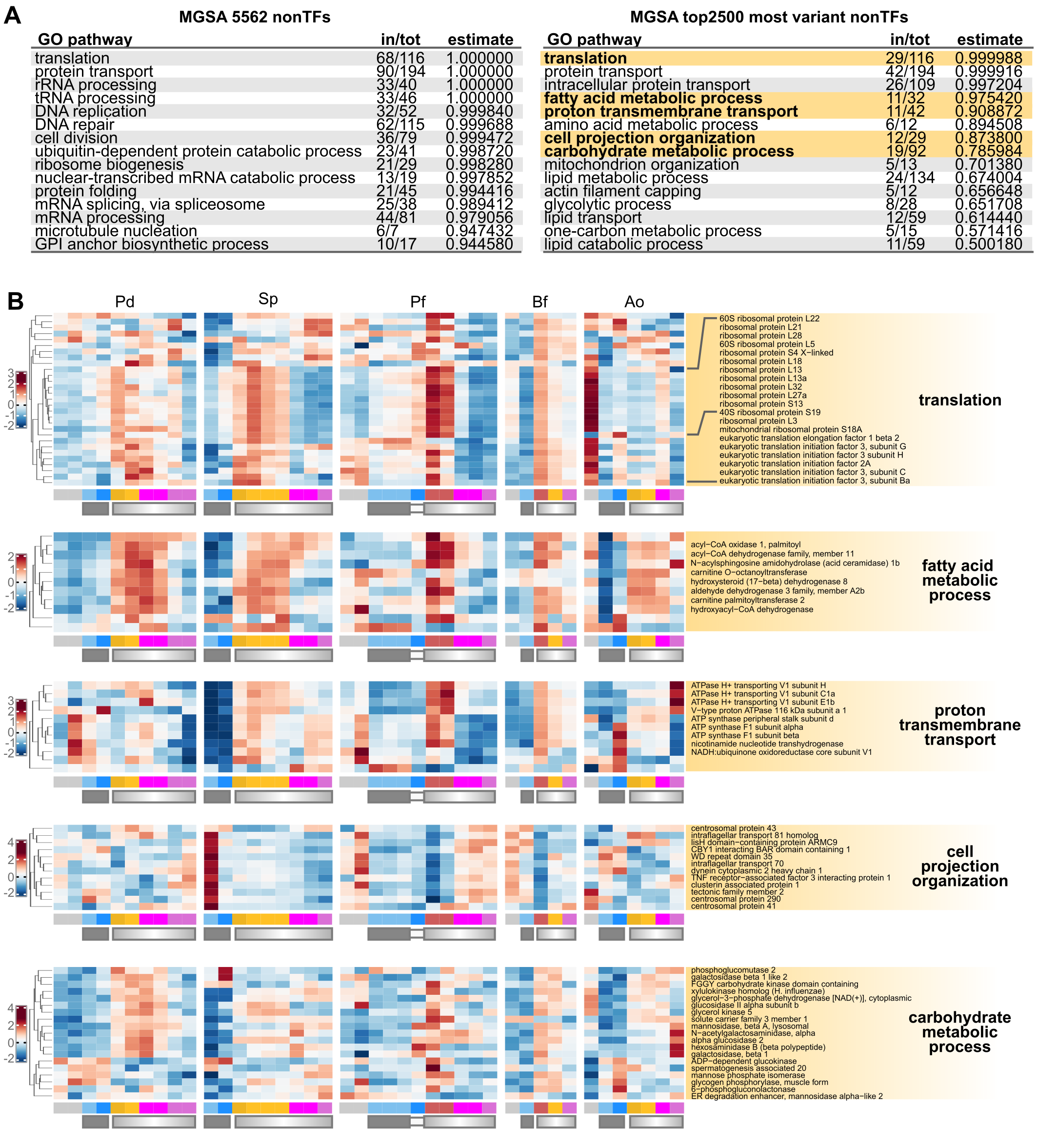

Supplement: S6 Fig — A) Summary results of enriched Gene Ontology pathways across all gut-expressed non-transcription factor genes (Left) and among the top 2,500 most variant subset of them (Right). Pathways names in bold and highlighted in yellow have their expression pattern plotted in B. “in/tot”: ratio between the number of genes of a given pathway present in the set considered, and the total number of genes belonging to that pathway; “estimate”: MGSA score. B) For each species, heatmap showing the expression pattern (z-scores) of all expressed genes belonging to the GO pathway of interest. Genes (rows) are clustered according to hierarchical clustering. Segments (columns) are ordered according to AP position. Solid and graded rectangles under the heatmaps indicate segments previously assigned to block and gradient gut compartments, as defined in previous sections. The data underlying this Figure can be found in https://doi.org/10.5281/zenodo.17746910. (PNG) [file pbio.3003571.s006.png]

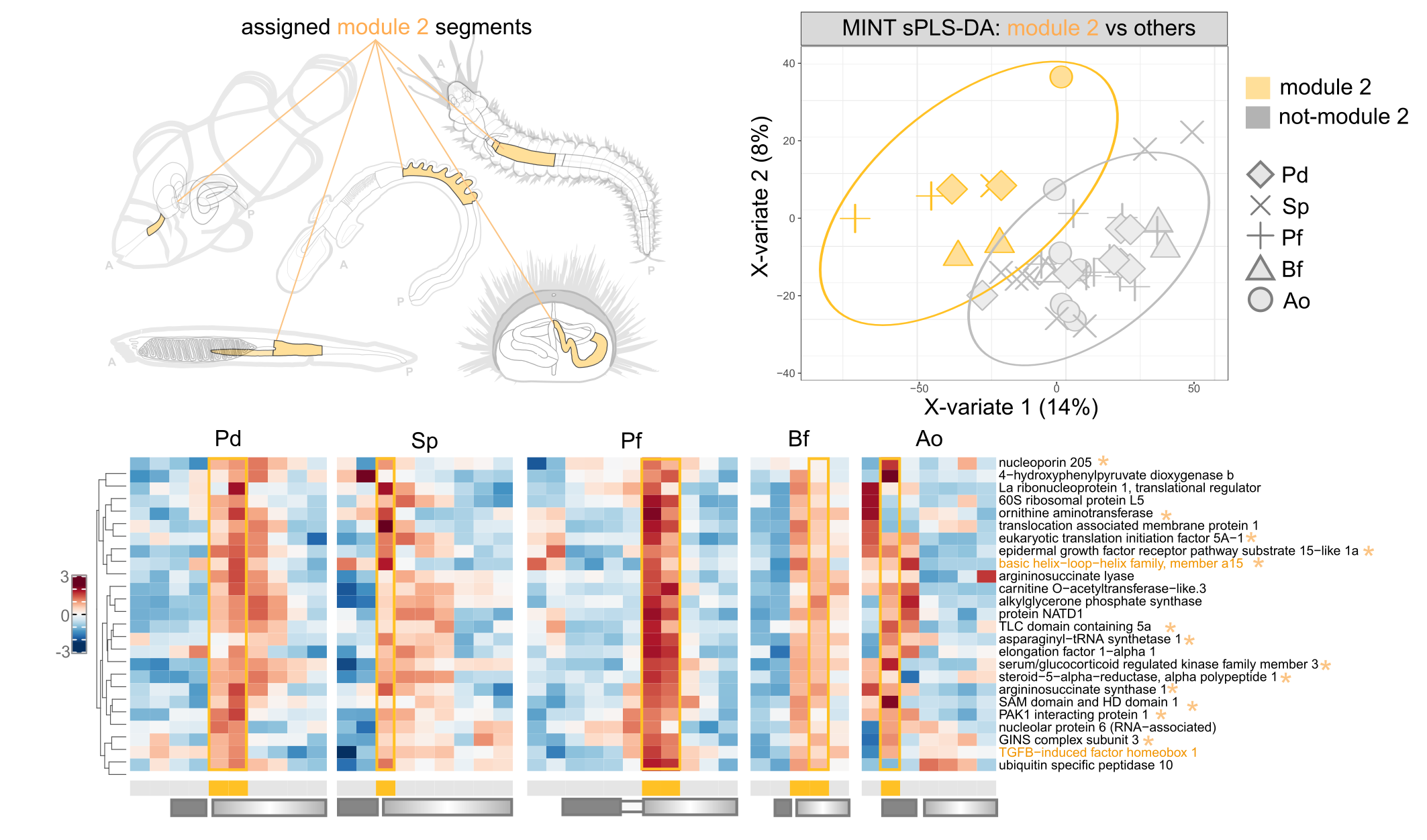

Supplement: S7 Fig — A) sPLS-DA of bilaterian gut segments marked by Module 2 TFs. Left: illustration of the segments to be discriminated (Module 2 segments). Right: separation of segments according to sPLS-DA discriminant genes. Bottom: For each species, heatmap showing the expression pattern (z-scores) of the top discriminant genes identified through sPLS-DA. Genes (rows) are clustered according to hierarchical clustering. Segments (columns) are ordered according to AP position. Solid and graded rectangles under the heatmaps indicate segments previously assigned to block and gradient gut compartments, as defined in previous sections. Gene names followed by an asterisk constitute the optimal sufficient discriminant set. Gene names in color are TFs. The data underlying this Figure can be found in https://doi.org/10.5281/zenodo.17746910. (PNG) [file pbio.3003571.s007.png]

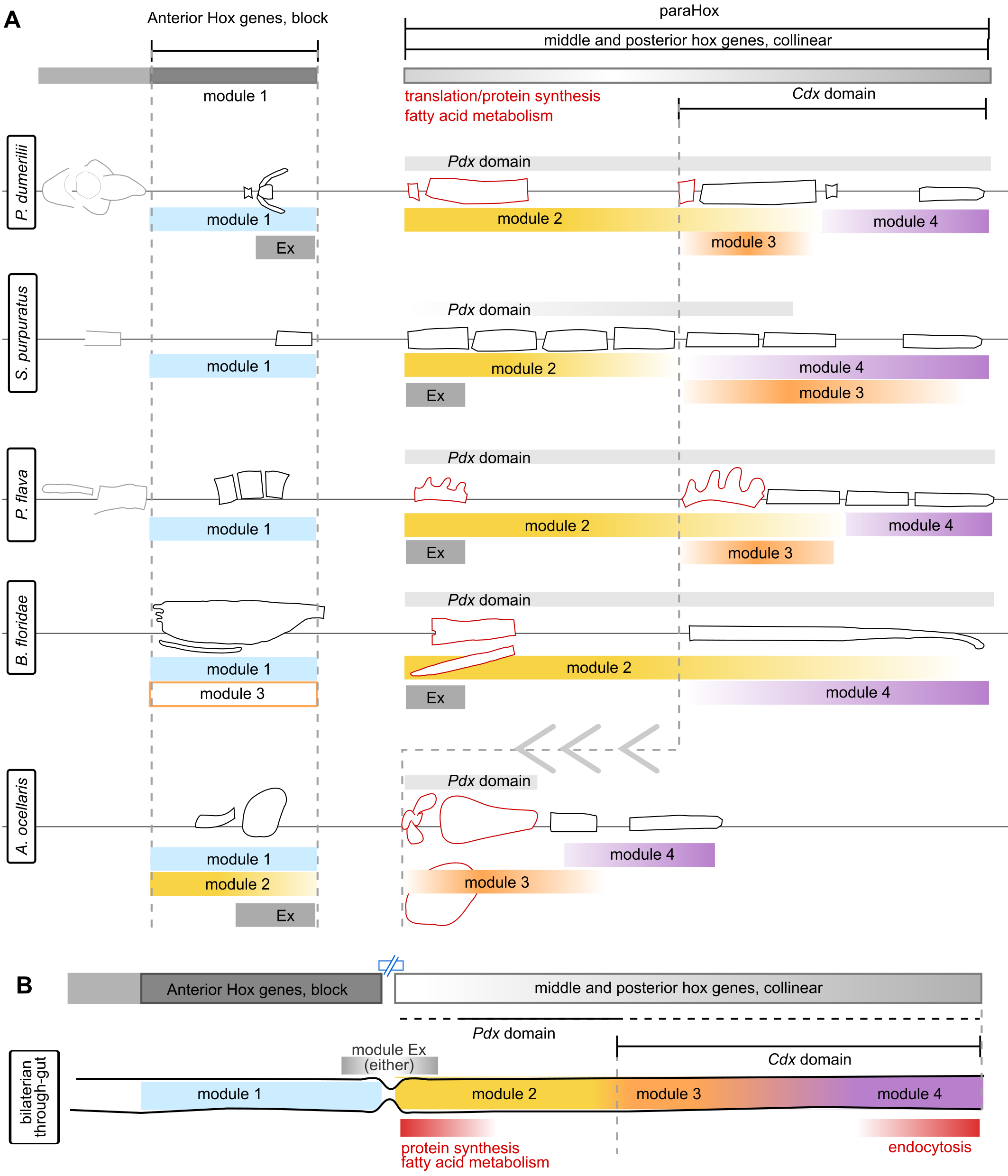

Supplement: S8 Fig — A) Summary of the AP arrangement of compartments, modules, Hox and ParaHox genes, and other common functions (red segments) in the adult through-gut of each of the five bilaterian species investigated. B) Summary model of the likely ancestral configuration of the adult bilaterian through-gut, based on the patterns observed in extant species. (PNG) [file pbio.3003571.s008.png]
