# Supplementary material for: Comparative transcriptomics reveal the common anteroposterior molecular blueprint of adult bilaterian guts
Source: PLoS Biol. 2026 Jan 5;24(1):e3003571. doi: 10.1371/journal.pbio.3003571 (PMC12768270; doi:10.1371/journal.pbio.3003571)
Supplement: Attachment — Submitted filename: PLOS_Vianelloetal2025AdultGut_ResponsetoReviewers.docx [file pbio.3003571.s011.docx]

**中央 中央研究院細胞與個體生物學研究所Institute of Cellular and Organismic Biology**

**Academia Sinica**

**128, Section 2, Academia Road**

**Nankang, Taipei 115, Taiwan, R.O.C.**

**Tel: 886-2-2789-9515**

**Fax: 886-2-2785-8059**

Dear Dr. Roberts, dear PLOS Biology Editorial Team,

We are pleased to submit the revised version of our manuscript “**Deconstructing the common anteroposterior organisation of adult bilaterian guts**”, and our detailed responses to each reviewer’s comments.

Most importantly, you will see that we have now significantly streamlined and restructured both the Results and the Discussion section. Following all reviewer’s requests, we have simplified the text, cut down marginal or speculative sections and figures, and clarified all flagged ambiguous points to improve accessibility and make the text much less heavy and easier to read to the broader reader. Our summary Fig 7 has now been edited to remove an important source of ambiguity reflected in several reviewers’ comments, and our Discussion section has now been heavily edited, streamlined, and re-structured, now much better highlighting the main take-away points of the study, and providing a much more balanced speculative section.

Within such editing work, we note the opposed suggestions of Reviewer 1 and Reviewer 3 regarding the amount of speculation within the Discussion section, and hope you will appreciate our taking both requests into account.

All presentational and textual improvements requested have been implemented, including the request to present absolute expression levels for key transcription factor panels, as well as our edits to improve the interpretability of the figures.

Hoping that you will find all of our responses satisfactory, we would like to thank you and the Academic Editor for your time handling our manuscript, and all reviewers for their extremely thoughtful engagement with our work, and for their precious feedback, which is now reflected in this improved version of the manuscript.

Our point-by-point answers to all reviewers’ comments follow.

With sincere thanks,

on behalf of all authors,

Stefano Vianello

## Response to Reviewers

Author responses in blue colour

**Reviewer #1 comments:**
[identifies himself as Chema Martin]

In "Deconstructing the common anteroposterior organisation of adult bilaterian guts", Vianello and co-authors investigate the anteroposterior patterning of bilaterian guts through bulk transcriptomic profiling. The evolution of a through-gut (a gut with a mouth and an anus) was, arguably, a transformative innovation that allowed the specialisation of the digestive system, potentially allowing the exploitation of new ecological niches and promoting speciation. Although most of the work has focused on investigating the formation and patterning of the through-gut in embryos and larvae, less is known about how adult bilaterian guts are patterned at the molecular level and whether that patterning is similar to that in embryos and conserved across lineages. To address this gap, the authors selected five representative species of more or less distantly related animal phyla (a vertebrate, an invertebrate chordate, a hemichordate, an echinoderm and an annelid) and dissected their adult guts into serial sections, which they then used to generate bulk transcriptomics in replicates. Applying sophisticated computational analyses, the authors discover that, despite significant transcriptomic differences, adult bilaterian guts are patterned into two primary regions (an anterior and a trunk/posterior region, primarily based on Hox gene expression) that can be further subdivided into four functional modules based on global transcriptomic signatures. Notably, genes commonly used to define endoderm/gut regions in bilaterian embryos and larvae are still deployed in the adults, generally maintaining the expected anteroposterior localisation based on the embryonic expression. This is seen even in the echinoderm, in which there is not a direct ontogenetic relationship between embryonic and adult guts. Overall, the manuscript is well-written, methodologies and results are clearly explained, and the observations support the main conclusions of the study.

I do not have major concerns about the analyses and approach, but I believe there are some points the authors may want to clarify:

We thank the reviewer for the time taken to read our manuscript, and for the very positive feedback on our work. We hope that our edits to the manuscript, and our comments below, are able to provide the clarifications needed.

- Most of the analyses are done at the level of z-scores, which is a good way to show and compare trends between genes, regions and species. However, it would also be meaningful to provide information on the actual expression levels, especially of transcription factors. For example, are Hox genes highly expressed in the adult gut? That is surprising, given that gene expression analyses in many invertebrates (at different life stages, including e.g. juveniles) have not detected a signal for these genes in the gut. The same applies to other TFs, as this would also help two align two seemingly contradictory results of the study: that gut transcriptomes differ significantly between species, yet there is some conserved signal, especially at the regulatory level (which, in all fairness, is not surprising).

We thank the reviewer for raising this very important point. We now include, for Hox panels (Fig 2), and Sp TF panels (Fig 3A), the number of counts (transcripts per million) for each gene plotted. To not overcrowd the figures, we only report the counts for the two segments with the lowest and highest z-scores: we provide the counts range for each gene. For panels where expression level labels would end up too small to read (Fig 3B), and for all other genes shown, we otherwise stress that counts data is fully consultable to interested readers at the data repository linked within our manuscript (<https://github.com/StefanoVianello/ASICOB_GCP_AdultBilaterianGuts>). Reflecting the changes we made to the figures, we added the following sentence to the Materials and Methods section (“Analysis of bulkRNAseq data” > “Data analysis and visualisation”): “*To provide a better understanding of the overall expression level and effective range of expression of selected genes along the AP axis, their highest and lowest heatmap z-score values were further overlayed with the corresponding absolute transcripts per million*”. The following was similarly added to the legends of Fig 2 and Fig 3: “*Overlayed numbers indicate the transcripts per million corresponding to the underlying z-score (highest and lowest)*”.

Between the most common choices of data to present in the heatmaps, we originally picked (gene-wise) z-scored values since our analysis focuses on anteroposterior patterning and therefore on *relative* differences in expression values across anteroposteriorly-ordered segments. As shared by the reviewer, this is the kind of understanding best facilitated by z-score heatmaps. We continue to not prioritise the absolute count data itself, at least not in terms of colour-coding, which we instead provide as a numerical overlay. This is mostly because of our investigative focus on relative expression differences, but also because counts-based heatmaps, if using a common scale, would put excessively high visual importance to highly expressed genes. We cannot confidently state that such genes are necessarily more important than lower expressed ones in terms of patterning value. Overall, we hope that the reviewer will agree that our new representation with colour-encoded z-score patterns, and numerically encoded count values best combines the two types of information within the context of our study.

Still, the counts data we now indicate need to be interpreted with respect to arguably subjective thresholds for which one could define a gene as being “expressed” or “not expressed”, “expressed high enough” or “not expressed enough” to have a role within the gut.

In our analysis, as we detail in a dedicated section of the Materials and Methods (“Analysis of bulkRNAseq data” > “Filtering and standard counts processing”), we adopted the classic arbitrary threshold of 10 counts to consider a gene as being expressed (enough). We later found that important TFs that were being filtered out by such a threshold (e.g. *Tbxt*/*Brachyury* and *Wnt10* in sea urchin) were still showing regionalised expression patterns, possibly suggesting that even at much lower expression levels such TFs may be biologically relevant. A post-hoc analysis we did not include in the final publication (and carried out after finding out that key TFs of prior interest had been filtered out) shows the number of TFs lost at increasingly stringent filtering threshold and finds our threshold quite suitable with respect to the overall distribution of TF expression. If anything, we found our threshold relatively conservative, and quite penalising for Bf. Future investigators of TFs function in adult guts may want to use our study as a prior to set less stringent filtering thresholds, something we however did not feel would be correct for us to do *post hoc*.

By maintaining our initial threshold of 10 counts, at the cost of not being able to consider a higher number of TFs (cfr. S2 Figure “d”: median loss of 21% of a species’ TFs, average loss: 23%), we therefore gain on confidence of the biological validity (non-noise) of the expression patterns of the TFs we do show in throughout the publication. In the cases were in situ hybridisation or other approaches have not detected these same TFs in adult gut samples, our data would indeed suggest that this is due to detection limits, that anteroposterior distribution differences do exist below such detection limits, that these are conserved, and that they therefore may be evolutionarily- or developmentally- relevant, and yet only accessible through omics or possibly single-molecule hybridisation approaches. Discrepancies with failed detection at juvenile stages would instead suggest that many of these genes are *upregulated* (and therefore specifically deployed) in the adult. Still, as now fully appreciable by the inclusion of the actual count data within key figure panels, and to answer the reviewer’s question, we can now directly show that the vast majority of Hox and TFs expressed in the gut are in fact highly expressed.

- It is more interesting that the foremost segment (mouth? oral ectoderm?) is more divergent and, in the summary figure, is left out (i.e., there is no module in the anteriormost part of the through-gut). Could the authors discuss the implications of this finding further? This contrasts with the observations about the hindgut, where there is more conservation. Does this have any evolutionary implications?

We thank the reviewer for providing us the opportunity to clarify this point. In our attempt to identify conserved anteroposterior patterns of expression among gut segments and seeing an overall high transcriptional divergence across all segments, we had to take a quite cautious, conservative approach. In Section/Fig 2 we find that the Hox/ParaHox AP patterning system does not extend to the foremost gut segments of many species (notably, Pd’s mouth and stomodeum, Pf’s stomochord and stomodeum), while it extends to the posteriormost segment of all of them. In other words, the “Hox register” of the gut does not deploy along its entire anteroposterior length. To simplify our follow-up comparative investigations, we therefore exclude Hox-negative segments from the comparative analysis. We take the Hox positive domain as the measure of the effective AP-axis of the gut, and we use segments within such span as the substrate of all comparisons.

In some sense, we needed to anchor our investigation of common AP patterning systems of adult bilaterian guts on as solid grounds as possible. Though we indeed pay the price of not being able to expand on such excluded segments within this publication, we do believe that focusing here only on segments that could at least have a comparative Hox basis was the most sensible choice. In this perspective, if we define as the anteriormost segments of the gut, those marked by the anteriormost Hox genes (i.e. the anteriormost segment of the ”gut Hox-AP axis”) rather than necessarily the anatomically anteriormost ones, then these are much more conserved than the hindgut, they are characterised by anterior Hox expression, Module 1 genes, and ciliary/Hh “functions”.

For our initial decision to root our analysis on segments that showed evidence to at least fall within the Hox measure of the gut, our search for conserved AP markers and conserved modules (Fig 4, Supp Figure 4x1 (now S3 Figure), Supp Figure 4x2 (now S4 Figure), Fig 5, Fig 6a, Fig 6b, Supp Figure 6x1 (now S7 Figure)) did not take anteriormost segments into account. This is also now made clearer from our more explicit annotation of gut segments in Fig 4, and Supplementary Figure4x2 (now S4 Figure) following another reviewer’s suggestions. The absence of anteriormost modules/markers, reflected in the Fig 7 summary, is similarly only a consequence of the fact these segments were excluded from the comparative search, rather than an indication that these are necessarily poorly-conserved segments or that no module would be found were they included. We therefore want to clarify here that we cannot conclude that “there is no module in the anteriormost part of the through-gut”, but rather, we did not consider (anatomically) anteriormost segment in our search for conserved modules, such that the question remains open and is not addressed by our paper.

This does not exclude future studies on the actual comparability of pre-oral/pre-anterior segments, though we want to further stress that this would have not been fully feasible anyways in our case since, for the clownfish, we were unable to collect an equivalent anteriormost/mouth domain that would be comparable (and that could potentially show to be Hox negative). We welcome such investigations, which are however something we could not properly carry on given our sample collection strategy, as well as already too dense of a manuscript.

- The discussion is long and very speculative. I do not think the authors' data provide new insights about the phylotypic stage question or the ancestrality of larval forms (actually, the alternative possibility, namely that an ancestral adult programme was deployed in the larvae as they evolved secondarily is not even considered and equally probable).

We thank the reviewer for the feedback. We indeed chose to write a long Discussion section that would also include speculative elements as well as tangential and intersecting themes in the field of gut evo-devo. This was deliberate, though we have now significantly restructured and streamlined the entire section in response to all reviewers’ comments. We have however also to point out the opposite feedback from Reviewer 3 on this specific section, in the sense they particularly appreciated the speculative Discussion, the discussion on the ancestrality of larval forms specifically, and in fact expressed a wish for overall additional speculation within the Discussion section.

We want perhaps to clarify that what our data brings forward is strong evidence for a much stronger continuity of gut expression patterns across metamorphosis than what generally assumed. The reviewer remains otherwise entirely correct in pointing out that such continuity could be in fact brought forward as supporting either argument about the ancestrality of the larval form, and we did not discuss the ”adult-first” scenario as an equally probable scenario given our data. We are aware of the open debate on these alternative possibilities and preferred not to go into it in this paper given that our data cannot formally exclude either, as correctly flagged by the reviewer.

To balance both reviewers’ feedback, we here i) keep the speculative paragraph but ii) have now edited it to provide a much more balanced picture that explicitly integrates the alternative (adult-first) hypothesis and better highlights the speculative nature of our statements. We have now also iii) significantly edited, shortened, and better structured the Discussion section, prioritising the exclusion of speculative sections, and have strengthened the explicit distinction between discussion and speculation.

For the specific section flagged by the reviewer here, the section now reads: “*Our results are particularly interesting in the context of the ancient discussion on the origin of larvae and the evolution of metamorphosis in bilaterians, a debate that has animated the literature since more than a century (reviewed in Gee, 1996). Our findings that embryonic patterning genes are redeployed in adult guts and maintain similar antero-posterior positioning highlight an unappreciated continuity across metamorphosis, even in cases characterised by dramatic morphological transformations, and bring forward a striking equivalence between larval and adult gut patterning systems. From a “larva-first” point of view, our findings suggest that metamorphosis does not erase the early program but rather reuses it within a new context. In that sense, metamorphosis could be seen not as a rupture, but as a reconfiguration of a conserved regulatory logic, inherited from the common bilaterian ancestor (Laudet, 2011). At the same time, our identification of adult bilaterian guts as AP-patterned systems (i.e. structures patterned by the same systems that are seen in larval guts) brings forward the alternative possibility: that what is deployed in larvae is an ancestral adult programme. Though our data cannot formally exclude either theory, our findings bring new elements to fuel this long-standing debate, specifically in highlighting the fact that bilaterian adult through-guts display an AP-patterning system mostly considered to be a larval exclusive.*”

The discussion about "the concept of the 'eigengene'" is confusing and is something definitely not mainstream in evo-devo.

We fully agree with the reviewer that the “eigengene” concept as a structural feature of animal spatial patterning systems is not (currently) a mainstream concept in evo-devo. We only came across it towards the end of our investigation, and as a result of a deliberate search for possible pre-existing conceptual frameworks onto which to anchor one of our main observations: the apparent counterintuitive conservation, in adult guts, of gene expression spatial *structure* rather than of the actual anteroposterior expression of the genes themselves (“*a cross-species conservation of global AP spatial expression structure, rather than relative AP localisation of most of the individual genes themselves*” (current page 19)).

At least based on our best interpretation of what was elaborated in (Kerstjens et al., 2024), their cross-species conservation of an “eigengene” (global spatial pattern of a set of genes) rather than of the domains of expression of the genes themselves within such pattern, is possibly the closest available formalisation in current literature. We quote here some of the main observations and conclusions of their publication, to try to highlight our interpreted closeness with our observations: “*We report that patterns of gene expression are remarkably conserved across ontogeny and phylogeny, even in the face of highly variable changes in the expression of individual genes.*”; “*First*, *spatial eigengene expression forms a static global pattern throughout development, despite the fact that individual gene expression is highly variable. Second, the eigengene measured in the mouse brain has a surprisingly similar spatial expression pattern in the larval zebrafish brain, despite a separation of more than 400 million years of evolution between the two species. Third, any random subset of about 50 genes shows largely the same spatial pattern in both mouse and zebrafish.*”.

It is in fact partly because of the fact that the “eigengene” concept is not mainstream, that we originally wanted to introduce it, if only by name, into our Discussion, as a conceptual tool maybe worth of increased consideration for interested readers. In other words, to possibly build further support for dedicated investigations of the validity of this concept across animal patterning systems, possibly specifically within evo-devo contexts.

Given that a more in-depth contextualisation of the concept would likely be too distracting from the main flow of the Discussion section, and that in its current form its mention remains confusing, we have now removed our reference to the “eigengene” concept from the manuscript. We removed the following sentence from the Discussion: " *We find the most resonant echo of such a property in the concept of the “eigengene” as a patterning feature, as elaborated in (Kerstjens et al., 2024).*”

Likewise, statements like "AP shifts of these modules with respect to the fixed compartments defined by Hox genes would in turn be at the origin of evolutionary innovations. We see such shifts to be characteristic of the chordate branch." are not entirely substantiated by the data, which is a single transcriptomic snapshot with low evolutionary resolution (how do the authors know that the transcriptomic differences are at the base of the morphological differences in the gut and is not the other way around?). I suggest the authors rework the discussion to focus on the few main take-home messages and their clear implications.

The reviewer is entirely correct, and we have now removed the indicated statement. Moreover, its corresponding paragraph within the Discussion has been significantly edited down, re-focusing this section. We hope that our otherwise newly reworked and streamlined Discussion section (see also modifications mentioned in previous comments) is now clearer and better able to highlight the main take-home messages from our investigation.

**Minor points:**
- Generally, throughout the manuscript (text and figures), gene names should be in italics.

Apologising for the formatting inconsistencies, we have now made sure that all gene names are italicised throughout the manuscript text and figures.

- In the discussion, the word adult is written in italics at some points. Is that on purpose?

The use of italics in the cases flagged was deliberate, and for added emphasis (“*adult*” = “adult and not embryonic”). We thank the reviewer for pointing out that this use may be ambiguous and have now brought back all instances of the word to standard format (current pages 19, 20).

- Figure 3: lacks a legend bar setting the range of z-scores in every panel. Annunziata et al is also in italics (not needed) but not gene names. Figure 4, 4x2, 5, 5x1, 6a, 6x, 6b: also lack legend bars for the heatmaps.

We have now added a z-score colour scale-bar to all heatmaps of the Figures indicated (Fig 3, Fig 4, supplementary figure 4x2 (now S4 Figure), Fig. 5, supplementary figure 5x1 (now S6 Figure), Fig 6a, supplementary figure 6x1 (now S7 Figure), Fig 6b), and apologise to all reviewers for their original absence.

The text “Annunziata et al., 2014” in Fig 3 has been de-formatted and brought back to roman as suggested. Formatting issues for all gene names have also been resolved as per the prior comment.

- Figure 7: in the top-left box below the A, there is a typo in gut (written as gutt)

We thank the reviewer for bringing this typo to our attention, which we have now corrected. We have also done the same correction to another instance of this typo, Supp. Figure 7x1B (now S8 Figure).

**Reviewer #2 comments:**

Vianello et al. present a molecular characterization of adult guts from five different species (P. dumerilii, S. purpuratus, P. flava, B. floridae, A. ocellaris) through anteroposteriorly-resolved bulk transcriptomics and cross-species comparisons to understand anteroposterior gene expression patterns and identify regional equivalences in gut segments across bilaterians, with the aim to unravel the evolution of gut patterning.

The data generated is of good quality, presenting a solid approach to dissect an interesting topic such as gut patterning. The authors critically analyze the data, often highlighting the weak points of their approach and justifying their choices in terms of data analysis, which is commendable. However, the paper is dense in information, which makes it challenging to read and follow the authors' ideas between paragraphs. Moreover, the terminology used is not always well-suited to the concepts being discussed. We suggest improving some of the results sections as said in the next points, simplify the text and perhaps keep only the figures showing the essential analysis needed to support the conclusions, since some of the graphs seem unnecessary. Overall, the paper is worthy of publication, even though some re-writing and a review from a bioinformatic expert is recommended.

We want to sincerely thank the reviewer for their time and for their thoughtful engagement with our work. We have overall modified the manuscript according to what was suggested, and hope that these changes were able to simplify the text, clarify and better adapt the terminology used, and improve the interpretation of key figures from the paper. Detailed answers to each of the reviewer’s comments follow.

**Major comments:**

The results should be presented with an easier style, especially in their technical aspects, to make the paper more understandable for a general public, such as the audience of PLOS Biology. Also, line numbers and page numbers are missing, making the review difficult.

We thank the reviewer for raising this point. The entire paper has now been simplified and streamlined, in response to all reviewers’ comments. We also sincerely apologise to all reviewers for the absence of page and line numbers in our original submission, and for the difficulties this created. Page and line numbers have bee now added in the revised version. We refer to these new page numbers in our responses below.

**Minor comments:**

Introduction, page 10: The authors should substitute:
 "in the last common ancestor" with "in their last common ancestor",
"through-gut" ancestor with "through-gutted" ancestor,
"on a developmental biology basis" with "from a developmental biology perspective"
Page 13. "deeply homologous structures" with "yet homologous structures".

We thank the reviewer for suggesting these changes, which improve our text and which we have now all implemented. Accordingly, we have substituted "*in the last common ancestor*" with "*in their last common ancestor*" (current page 2), "*through-gut*" ancestor with "*through-gutted*" ancestor (current page 2, but also in two other occurrences page 4 and 20), "*on a developmental biology basis"* with *"from a developmental biology perspective*" (current page 2), and "*deeply homologous structures*" with "*yet homologous structures*" (current page 4).

Results, Section 1, page 14: In this paragraph, the authors multiple times refer to anterior or posterior compartments without specifying the names of tracts that are a part of such compartments, e.g., "we note that i) anteriormost gut segments", " ii) segments posterior to this first domain". It would be better to clarify which gut segments (name the most anterior and posterior ones) form these molecular compartments.

As suggested, we have now supplemented this section with an indication of the actual segments being referred to. We now additionally explicitly refer to the corresponding graphical indication of the segments within each compartment of Fig 1B (grey and blue lines under numbered segment groups). We thank the reviewer for bringing this point to our attention.

The section (current page 5) now reads: “*Specifically, we note that anteriormost gut segments (Fig 1B grey lines; Pd mouth to oesophagus, Sp stomach-1 to stomach-4, Pf pharynx-1 to pharynx-3, Bf endostyle and gills, Ao oesophagus to stomach) can consistently be summarised as a single well-defined molecular compartment, which excludes expected highly-dissimilar pre-oral segments, see the case in Pf. Concomitantly, we observe that segments posterior to this first domain, up to the terminal most end of the gut, define a second compartment of graded AP similarity where transcriptional similarity decays with distance (Fig 1B light-blue lines; Pd transition-2 to hindgut, Sp intestine-1 to intestine-3, Pf hepatic-1 to intestine-3, Bf liver to hindgut, Ao pyloric caeca to posterior intestine).*”

Results, Section 3, page 22: "embrionic-" is "embryonic", remove "-"

We thank the reviewer for bringing this suspended hyphen mistake to our attention, now corrected as suggested (current page 10)

Results, Section 3, Figure 3A-B: add color legend directly in the figure to make visualization easier, e.g. red/blue for expression levels (with values, where possible) and blue = foregut, yellow = midgut etc..

Thanking the reviewer for the suggestion, we have now directly added a colour legend to Fig 3 indicating both gene expression values (qualitative, for our representation of the Annunziata et al., 2014 larval data, and quantitative for the adult gut z-scores) and gut segments (blue= foregut, yellow= midgut, magenta = hindgut; as defined in the larval gut and as inferred in the adult gut based on larval patterns).

Results, Section 4, Figure 4D, substitute "to not match" with "not to match". The red squares underneath the heatmaps indicate the gut segments described in Figure 1, but even in this case it would be useful to write a legend to recall the reader of such fragments.

We thank the reviewer for highlighting the sentence construction error in current page 13, which we have now corrected as suggested (substituted "*to not match*" with "*not to match*").

We have also now modified panel Fig 4D as suggested, adding a legend to each colour annotation, and overall clarifying what segment each square represent by adding their corresponding number (assigned in Fig 1, but also already re-introduced in an earlier panel of Fig 4D, panel 4B). We have done the same change to another occurrence of this same situation, Supp. Figure 4x2 (now S4 Figure) D. This significantly increases the interpretability of the figure(s), for which we thank the reviewer.

Results, Section 4, Supp. Figure 4x1: missing dot at the end of the sentence.

We thank the reviewer for bringing this omission to our attention, the dot has now been added (figure now called S3 Figure).

Results, Section 5, Figure 5A: a legend with expression values is missing.
Results, Section 5, Supp. Figure 5x1B: a legend with expression values is missing.
Results, Section 6, Figure 6: a legend with expression values is missing.

We have now added a z-score colour scale-bar to all heatmaps of the Figures indicated (Fig 5, supplementary figure 5x1 (now S6 Figure), Fig 6a, Fig 6b, supplementary figure 6x1 (now S7 Figure)) and apologise to all reviewers for their original absence.

Page 15: the authors propose the "transition sphincter" as the constriction between the "block" and "gradient" components of an adult gut. At page 45, in Figure 7, they specify that the transition sphincter in adult bilaterian guts is located right anteriorly the Pdx+ domain, and they color the sea urchin larva's cartoon accordingly. Having to make a comparison between larva and adult, this "transition sphincter" would rather correspond to the so-called larval "cardiac sphincter" (the constriction between esophagus and stomach). If this is correct, in larvae (at least S. purpuratus 72hpf plutei) there is no Pdx domain right after that sphincter; Pdx1 transcripts are present only in correspondence of the pyloric sphincter, while Pdx1 protein expands up to mid-stomach, not in the complete stomach.

We are sincerely thankful to the reviewer for highlighting these clear issues with the interpretability of Fig 7. We first and foremost want to confirm that we indeed defined the transition sphincter as the transition between block and gradient domains, and therefore mainly also identify it as the sphincter marking the posterior boundary of anterior Hox gene expression within the gut. This is the vertebrate pyloric sphincter, transition between the vertebrate stomach and the anterior intestine. In adult invertebrates, it is the sphincter transitioning between oesophagus and invertebrate stomach. In terms of modules, it is the sphincter consistently marking the posterior end of Module 1.

We also want to confirm that, indeed, having to make a comparison with the sea urchin larva, it would correspond to the cardiac sphincter. In this sense the colour coding (transition between Module 1, light blue, and Module 2, yellow) is correct and consistent between larval and adult configuration.

We instead do not find *Pdx* as being a consistent match or a reliable marker for this sphincter (specifically because of the observed adult sea urchin configuration). We cannot use *Pdx* as a marker of the transition sphincter for the very inconsistency flagged by the reviewer, and which we find to also apply to the adult. The main cause of confusion in the summary figure therefore appears to come from the way we indicated e.g. “*Pdx* domain” in the top summary diagram of Fig 7A. What the “|-- *Pdx*--” indication meant to symbolise, was that the anterior border of adult *Pdx* expression falls within the indicated area (in practice, the area where module typically 2 is): a *range* where *Pdx* is consistently found to be expressed in adult guts, rather than to necessarily imply that in all cases it reaches all the way up to the transition sphincter. In this interpretation, it also reflects the situation of the adult Sp. Though we detect *Pdx* transcripts in all stomach segments (including stomach-1, see Fig 2), they are certainly uniquely concentrated to the posteriormost segments of Module 2 (i.e. the stomach side of the adult sea urchin pyloric sphincter) rather than being already robustly high at the cardiac end (transition sphincter).

We thank the reviewer for identifying this ambiguous notation and have now edited the symbols used in the topmost diagram of Fig 7A in an attempt to indicate that the *Pdx* range need not to extend all the way up to the transition sphincter. In Supp. Figure 7x1 (now S8 Figure), which allows us to detail species-specific configurations, we now also replaced the solid shading indicating the domain of *Pdx* expression with a graded shading that better represent the posteriorized-shift of this marker within that domain. We instead keep the larval colour-coding unchanged since colours in this figure are not meant to indicate ParaHox genes, but only overall Modules. Still, we faded out the colour-coding in the larval cartoon (as well as in the ancestor cartoon), to properly indicate that these “projections” of our adult findings are speculative and will remain so until actual experimental verifications, which we welcome.

Removing this ambiguous notation, the adult sea urchin *Pdx* expression in fact matches the larval configuration described by the reviewer, with a *Pdx* expression profile high at the pyloric sphincter rather than the cardiac sphincter, giving us further confidence on the internal consistency of our model. We thank the reviewer again for alerting us to the ambiguity in this figure, which resulted in very helpful amendments.

Moreover, if once again this interpretation is correct, this figure is also misleading for the "Ex" domain: in the sea urchin larva, the exocrine domain is not present at the end of the "block" component. Therefore, for the figure not to be misleading, this reviewer suggests correcting the colors in the sea urchin larva cartoon, taking into consideration such differences.

Our graphical encoding for module “Ex” in Fig 7A, a grey domain split at either end of the transition sphincter, was meant to represent a range of possibilities: that it is found at the posteriormost end of the block compartment, OR that it is found at the anteriormost end of the gradient compartment. This is to reflect what we summarise in the text as “*In Pd and Ao, [the Ex module] is detected in posterior segments of the block compartment (i.e. oesophageal segments, including the vertebrate stomach; i.e. overlapping module 1 segments), in the other species [the sea urchin case for example] it is detected at the anteriormost segments of the gradient compartment, i.e. overlapping with module 2 segments.*”

The Sp adult gut is accordingly the case where the Ex module, in its relation to the transition sphincter, is at the anterior end of the gradient compartment (mostly in the expression of *bhlha15/mist* and *ptf1a*). In other words, in the adult too, the exocrine domain is not at the posterior end of the block compartment, as flagged by the reviewer for the larval condition. The Ex-segment in the sea urchin gut would be in the segment we called “stomach 1” in the adult, colour coded in yellow alongside other Module 2 segments. In turn, we infer “stomach 1” to be the adult match to the anterior end of the sea urchin larval stomach, also colour-coded in yellow. This anterior area, abutting the transition sphincter (larval cardiac sphincter), is indeed where we understand exocrine cells to actually be located in the larva (the sea urchin acinar-like cells of Paganos et al., 2022).

In summary, and if our interpretation is correct, there has been a misunderstanding from the interpretation of our “split” module Ex icon to imply that the Ex-module would be located in both positions in all species. It symbolises instead a summary of alternative possibilities seen in different species. To increase the interpretability of our summary figure (7A) on this point, we now more explicitly indicate the split icon of the Ex module (added the indication “*either*”) and further report the exact position of each module in each cartoon in Supp Figure 7x1 (now S8 Figure). There is therefore in fact a match between the expression patterns we see in the adult, and those known in the larva.

We apologise to the reviewer for the confusion caused by our pictogram choice and thank them for their important assistance on improving the clarity of our summary figure.

Figure 7: Similarly to what described above, the anterior border of Pdx1 domain in panel A does not appear correct for sea urchin adults, because not in agreement with what reported in the heat map shown in Figure 3A. Therefore, also the color code reported for the sea urchin adult gut appears misleading.

The colours we use in all cartoons in Fig 7 only represent the position of the Modules (1: light blue, 2: yellow, 3: orange, 4: purple), which we find, yes, correlated to ParaHox expression domains, but quite flexibly so, and especially for *Pdx*. We thank the reviewer for already highlighting the ambiguity about our *Pdx* notation, which we have now addressed by modifying the Figure as per their previous comment (we did not intend to imply that *Pdx* always extends to the transition sphincter). Still, and for what concerns the colour-coding/modules specifically, these remain correct in the sense that Module 2 markers do in majority mark adult stomach segments (stomach 1 to 4) despite a characteristic posterior bias of several of them towards the adult sea urchin pyloric sphincter (Fig 4D).

In the Results, multiple times the authors refer to sPLS-DA and they write, at page 37: "Critically, our module-based interpretative framework allows us to impute shared segment identities across species, and therefore to apply supervised dimensionality reduction approaches such as Partial Least Squares - Discriminant Analysis (sPLS-DA, (Rohart et al., 2017)) to identify their discriminating markers and/or expand the signatures beyond TFs alone". For non-expert readers, could the authors add a brief justification, in the main text, of why this method is more suitable to the data they present? The same comment can be made for R2E representation.

Anticipating the need to better contextualise these two techniques, we had already allotted dedicated sections for each of them in the Materials and Methods section (“sPLS-DA”, and “Rank 2 Elliptical (R2E) seriation”). These sections better detail each of the methods but indeed do not significantly expand on the rationale behind their choice (except maybe for R2E, where this is already the case). Briefly, for what concerns sPLS-DA, our comparative transcriptomics analysis follows, in big part, the approach modelled in Mantica et al., 2024, who use this technique to “*to isolate the orthogroups with the most distinctive expression profiles in each tissue (compared with the others) across all species*”. Crucially, this is immediately possible when tissues equivalences are known (e.g. all brains of different species, all muscles of different species), which ensures that the markers identified through sPLS-DA will be biologically grounded and informative. However, this technique could not be immediately deployed in our study, since the identification of equivalent gut segments was in fact one of the main tasks we needed to accomplish. We stress that, by design, sPLS-DA can find common markers to any arbitrary combination of tissues, even where these tissues are not in fact equivalent, and the value of its application, in terms of biological insight, is therefore dependent on the actual biological equivalence between the segments chosen. It is for this reason that we first deploy unbiased, generalised explorative and classification approaches, and only then, having found e.g. Hox coordinates and TF modules to ground our inferences of tissue equivalences, we (can) use sPLS-DA to find discriminating markers of such segments.

Our eventual choice of R2E seriation is instead justified by the fact that current standard transcriptomic investigations of spatially or temporally auto-correlated data (i.e. patterning or time-series designs, in fact extremely common in (EcoEvo)Devo studies) usually rely, for exploratory and visualisation purposes, on i) clustering of the genes, and ii) heatmaps of these clusters. To highlight spatially- or temporally-progressing trends, these clusters are usually manually re-ordered so that clusters whose genes peak early on are flipped to the top, and clusters whose genes peak at the end of the space/time-series are flipped to the bottom. Though this can be semi-automatised, we still dedicated a lot of time and separate effort to identify less subjective, and more reproducible seriation (ordering) criteria, adapted to the very structure of the data, and in fact designed for such kind of data. R2E is specifically such an example, and we detail its technical specifications within the dedicated section of the Materials and Methods. In fact, we hope that our introduction of this seriation criterion may help push towards its more widespread use in the EvoDevo bulk transcriptomics field, and possibly in turn more qualified appreciations of its advantages and limitations with respect to traditional and alternative visualisation approaches.

From previous iterations of this manuscript, in which we were in fact including such justifications in the main text, we noted that even if in their abridged versions they were ending up too cumbersome to the overall flow. For this reason, they ended up being left out. We have now added them back, but in their dedicated Materials and Methods subsections. Correspondingly, we now refer readers to such dedicated sections upon first mention of either technique within the main text.

Supplementary Figure 2x1 seems not to be essential for the general message of the paper, but can be relevant for clownfish specialists and Hox genes evolution-experts. If the authors want to keep such a figure, perhaps they could expand the paragraph in which they explain its meaning.

We thank the reviewer for bringing this to our attention, and indeed agree that these alternative visualisations may be of specialised interest only (hence our initial choice of relegating them to the Supplementary Figures). We fear that any further in-text elaboration on the type of insight facilitated by these alternative heatmaps would further burden the flow of the main manuscript. In the context of an overall simplification of the manuscript, welcomed by all reviewers and as suggested, we have accordingly removed Supplementary Figure 2x1. We are comforted in remarking that specialist readers, if interested, would still be able to refer to these alternative visualisations in the preprinted version of this work.

Along with the figure, we have also removed its corresponding reference within the main text: “*Due to the presence of 7 different Hox clusters in clownfish, we order Hox genes in a way that reconstructs their AP sequence of expression (R2E seriation, and which indeed overall matches paralogue numbers), but we also provide cluster-specific maps (ordered by paralogue number), and paralogue-specific maps (ordered by cluster) in Supplementary Figure 2x1 for more convenient reference.*”

Page 22: "We show that the expression of all of these early patterning markers (including, here again, Hox and ParaHox genes) is conserved along the Strongylocentrotus purpuratus adult gut, virtually unchanged (Figure 3A)". The authors should also cite here the paper from Paganos et al., 2025, where the expression of the gut patterning genes was explored through snRNA-seq of a post-metamorphic stage (juvenile) of Paracentrotus lividus.

As recommended by the reviewer, we now cite the paper from Paganos et al., 2025 also in the indicated section of the Results. The section quoted by the reviewer (current page 9) now follows with the sentence: “*Such conservation has also most recently been shown in the post-metamorphic (juvenile) gut of a close species, Paracentrotus lividus (Paganos et al., 2025).*”.

The work from Paganos et al., 2025 otherwise (further) features in the Discussion section, as was the case in the original submission. “*To this point, studies that have most recently directly addressed the bridge between gene expression patterns before and after metamorphosis, are indeed cementing the re-deployment of embryonic and larval molecular signatures into juvenile life as a feature of most cell types, including those of the gut ((Paganos et al., 2025), Paracentrotus lividus).*”, current page 18.

Page 22: "We find this conservation extraordinary, not only because it suggests the long-term permanence of a "larval" AP gut signature long into adulthood, but also because such signature is manifestly retained even across drastic ontogenic restructuring of the very physical materiality of the gut tube itself, as is the case of what happens specifically to the sea urchin gut tube during metamorphosis (Holland, 2020)". This sentence does not read well. Moreover, it could be more correct to propose that the regulatory program used in larval gut development is reused after metamorphosis for the patterning of the adult gut. The authors should also take into account that a part of the larval gut is retained during and after metamorphosis, becoming a part of the adult gut.

We have now rewritten the sentence and thank the reviewer for flagging its clunkiness.

We also thank the reviewer for pointing out the very likely alternative possibility: a re-deployment of the larval regulatory programmes rather than a maintenance of the patterned domains themselves across metamorphosis. We were very deliberate in always putting forward both possibilities in all other parts of the main text and Discussion, and this instance had escaped us.

According to the reviewer’s suggestions, the simplified, split, and edited sentence (current page 9) now reads: “*We find this conservation extraordinary as it suggests the long-term permanence or re-establishment of a “larval” AP gut signature long into adulthood. Furthermore, such signature is manifestly retained/redeployed even across a drastic ontogenic restructuring of the gut tube itself, as is the case of what happens specifically to the sea urchin gut tube during metamorphosis (Holland, 2020)*.”

We hope the reviewer will accept our use of the term “restructuring” (maintained from the original version) as our best attempt to convey that this is not a total discontinuity but involves partial maintenance of the larval gut through metamorphosis. Earlier versions of this section, with more dedicated accounts to the extent of loss/maintenance of different larval gut regions, were eventually sacrificed for reasons of length, and to simplify the overall flow within the Results.

Page 22 "In other words, a subset of anteriormost or posteriormost gut markers, which would appear as having poor AP conservation, may be best understood at a more holistic pan-bilaterian level as "terminal" markers (see e.g. foxD, foxP, foxI, Figure 3B), and be conserved in this role across life stages and species". The word "terminal" is a bit misleading, as it is usually referring to terminal differentiation genes or to the end of a structure. The authors should find another way to describe them for their position in the stomach or cluster. Figure 3B allows for better elaboration. Instead of having a vague discussion of the "terminal" markers, they should modify this section properly, describing in detail what they see in every species and put it into an evolutionary scenario.

We thank the reviewer for highlighting the ambiguity introduced by our choice of the word “terminal” to describe the expression pattern and the markers in question. Indeed, this choice had been the subject of significant discussion and consideration even among us authors, resulting in “terminal” as a compromise. We are very grateful for the feedback and return from colleagues for this initial “pressure test” of the term.

To first and foremost clarify, our use of the term “terminal” is to be understood as a reference to the expression patterns of the *Drosophila* “terminal system” genes, with in situ hybridisation patterns of classical “terminal” genes tailless (*tll*) and huckebein (*hkb*) distinctively marking “terminal” expression at both termini of the embryo, and arguably of not-too-distant recall in the imaginary of an EvoDevo/AP-patterning readership. In this sense, we do indeed use “terminal” to mean genes expressed “at the end of a structure” (i.e. the anteriormost and/or posteriormost segments we considered). We do believe that this acceptation of the term is particularly fitting in qualifying genes such as *foxD*, *foxP*, *foxI* here, that in some species are “anterior” markers, in other species are “posterior” markers, in other more are “posterior and anterior”, and whose conservation at the panbilaterian level would therefore better be identified as “makers of the termini” (either or both). This is to differentiate these genes from genes such as *six3/6* or *Hox13* (to keep referencing Fig 3B), that are consistently always at the same terminus of the gut in all species and are therefore fully “anterior” or “posterior” markers. Just to further clarify, the genes we define as terminal would not include those that show expression in the (invertebrate) stomach only, but rather specific genes found in the foregut and/or the hindgut.

Still, as pointed out by the reviewer, the ambiguity and overlap with “terminal (differentiation)” remains (especially since we use it ourselves in this acceptation in the Discussion, current page 19: “*That is, and reversing more intuitive embryo-based perspectives, could embryonic gut patterning genes be genes with key adult functions as regulators of key adult endodermal functions, gut “terminal selectors”*?”).

To remove any ambiguity, indeed also highlighted by other reviewers, we now much better describe our use/definition of the word upon first mention and further disambiguated our use of “terminal” in its “terminal differentiation” acceptation later in the text. Accordingly, the section highlighted by the reviewer has now been amended to add: “ “*Terminal” markers, in bilaterian through-guts, would therefore be markers that in some species are “anterior” markers, in some species are “posterior” markers, and in others more are “anterior and posterior” markers. At the pan-bilaterian level, they are “markers of the termini (anterior gut and hindgut)”, across species or across life-stages.*” Correspondingly, our late use of “terminal selector” has been disambiguated and now reds: “*(using here “terminal” in its meaning of “terminal differentiation”)*”.

We also would like to highlight that this shared-belonging and conservation within a “terminal” class is precisely the evolutionary scenario that we foresee. That is, we do not interpret such observed anterior/posterior shifts as an evolutionary shift or a trend, or as a necessary indication of protostomous/deuterostomous/amphistomous developmental origin of these guts, but rather as the evidence for an inherent alternative deployability of these genes at either terminus. In our view, this in fact reconciles the fact that many foregut and hindgut markers are seen at the opposite pole across larvae of different species, that this is the case also for the adult gut (cf. also our expansion on *Tbxt* in response to a later comment), and that these are the same that we see shifting even across ontogeny within the same species, as we can at least see in sea urchin. In this sense, we define “terminal genes” as belonging to a separate conceptual class than “posterior” and “anterior” gut markers, but which can only be highlighted by cross-species, and cross-stages approaches.

Page 25: remove "de facto", substitute with "as transcription factors".

The sentence has been simplified as indicated (removed "*de facto*", current page 10). We thank the reviewer for the suggestion.

Page 26: "deconstruct" instead of "decompose".

The word has been substituted as indicated ("*deconstruct*" instead of "*decompose*", current page 11)

Brachyury is one of the most conserved markers for the posterior gut in many animals, it is also present in (Annunziata et al., 2014). Why is it not mentioned in the paper and excluded from the analysis?

We thank the reviewer for raising this very important point, which gives us the opportunity to expand on a result of our analysis which we found particularly interesting but that could not find a place within our (already information-dense) final manuscript.

As indicated by the reviewer, *Brachyury* is one of the most conserved markers for the posterior (and posterior gut) in many animals, certainly at early developmental stages. We include it in the final paper for its membership in the (Annunziata et al., 2014) set of transcription factors (*Tbxt*, Fig 3A) and yet could not elaborate on it further. This is unfortunately because, as indicated by the truncated dashed line in Fig 3A, *Tbxt* is not* expressed in adult sea urchin gut tissues (*it is expressed at levels that fell below our filtering threshold). In turn, the absence of expression of *Tbxt* in adult Sp gut samples disqualifies an analysis of *Tbxt* expression in any of the other four species, even though these species do express *Tbxt* in their guts (Sp will be missing the *Tbxt* orthogroup entry > the *Tbxt* orthogroup will not be classified as a shared bilaterian gut-expressed orthogroup > *Tbxt* orthologues will be discarded from the final merged expression matrix). This is the reason *Tbxt* does not and cannot come up as a marker of any of the modules, or as an AP-associated TF in any of the cross-species approaches we have undertaken.

Anticipating an interest in the expression pattern of *Tbxt*, an interest we ourselves shared, we deliberately report this circumstance in a dedicated section of our “Materials and Methods” > “Analysis of bulkRNAseq data” > “Filtering and standard counts processing” (current page 28): “*We note that the threshold of 10 counts, chosen a priori based on usual practices, resulted in the filtering out of key genes of interest (e.g. Wnt10 and* ***Tbxt*** *in Sp, Cdx4 in Ao). Though we later noticed that the pattern of expression of many filtered-out genes of interest is in fact coherent with expectation based on published adult gut literature or on known embryonic patterns, we preferred to prioritise robustness of results over expansion of the genes included in the comparison. We maintained the original count threshold set a priori*.”

A targeted investigation of *Tbxt* expression patterns requires, *post hoc*, a lowering of the threshold used to consider a gene as “expressed” (see also response to Reviewer 1 comment). The results of such analysis are reported below, which would however need to be heavily disclaimed for its reliance on low counts numbers. As can be seen in the results above, *Tbxt* (NCBI LOC576774) would be shown to mark the pharynx in the adult sea urchin gut, therefore demonstrating a drastic shift of termini compared to its larval pattern.

The adult gut of the other four species under investigation does express high levels of *Tbxt*. In *Platynereis* it is expressed at the anterior (mouth), in acorn worm and in amphioxus it is expressed at the posterior, in clownfish it is expressed at the anterior and posterior.

In fact, we do believe that our *Tbxt* data strengthens one of the recurring patterning principles we describe in our paper and reveals fundamental and surprising features of this gene and its role in gut patterning in general. The very adult maintenance of posterior *Tbxt* expression, even just in our vertebrate species, is not part of the mainstream understanding of this marker, archetype of vertebrate embryogenesis.

Referring to our previous comment on the distinctive “terminal gene” class, this would make *Tbxt* such a marker: not necessarily to be understood as markers of the posterior and of the anterior per se, but rather as general markers of either terminus, and in this aspect found at a different end of the guts of different species, at both ends of others, and in fact potentially shifting ends during ontogeny of a single species.

We did not include any of these results in the final version of our paper for the reasons cited above, privileging a maybe more understated and cautious final paper (which would consider *Tbxt* as not expressed in sea urchin adult guts) even if meaning having to abstain from very interesting speculative conclusions from key, but low-expressed, patterning markers. We are comforted to know that future investigators that may more robustly recover *Tbxt* as an adult gut patterning marker across bilaterians, may reference our work and use the raw data we make available with this publication as further support for this thesis.

Page 27: "This pattern echoes our previous recovery, at the species level, of a shared "terminal" pattern of gene expression, a conservation of gene expression at either terminus, and ontogenetic inter-termini shifts of gene expression at least in sea urchin." Same comment for "terminal" at page 22. The authors should rephrase it.

Following from the previous comment on the use of “terminal”, we note that we have now much better clarified our use of the word “terminal”. Nonetheless, we have now rephrased the sentence (current page 12). We thank once again the reviewer for the precious feedback.

The word “terminal” has been eliminated and the sentence now reads: “*This pattern echoes our previous recovery* *of anterior or posterior TFs in some species as markers of the opposite terminus (or of both) in other species, and ontogenetic inter-termini shifts of gene expression at least in sea urchin*”

**Reviewer #3 comments:**

The authors investigated the transcriptomic profile of five bilaterian species along its AP axis, one protostome (the annelid Platynereis dumerilii) and four deuterostomes. They found that all species show an anterior block domain followed by a posterior gradient domain, verified by two bioinformatic approaches, PCA and heatmap. The most likely organizer genes of this AP pattern are hox genes and indeed they find in all species that the hox genes show a colinear expression even if the Hox cluster itself has been disrupted in the genome. Importantly, they find (and partially confirm) that among the Parahox genes, gsx is barely expressed in the gut, while pdx and cdx consistently pattern the gradient domain of the bilaterian guts. They also show that the conservation of gene expression pattern is also preserved for a list of transcription factors that were shown previously having a graded expression in the gut of the sea urchin (Annunziata et al., 2014). Next they analysed all non-TF genes, which represent largely the effector and structural genes of the gut. Through the application of various bioinformatic tools, Again, they find fairly good conservation of AP patterning across species, especially between Platynereis and the two ambulacranian species. The two chordate species display significant rearrangements of the modules suggesting that these represent chordate innovations, consistent with other molecular and morphological evolutionary changes observed in vertebrates. Given that the investigated species comprise protostomes and deuterostomes, this implies that this AP patterning system is an ancestral bilaterian trait. Remarkably, the authors find that much of the conserved molecular features are derived from maintenance of well conserved and studied embryonic regulators. This is intriguing given the drastic metamorphosis from a larva to the adult involving complete remodelling of most tissues. This strongly suggests that embryonic patterning genes are redeployed in adult guts and maintain similar antero-posterior positioning, which has important implications on the long-standing question of whether the larva is ancestral or a secondary integration or whether the adult is an addition to a former life stage.

I find the study very well designed through the choice of the selected species (although a second protostome would have been desirable). While the experimental procedure is straight forward, the authors have undertaken major emphasis on the bioinformatic analyses of the data, testing and evaluating multiple ways to extract correlations of spatial gene expression patterns. This is done in a thorough and careful manner. The data represent an important resource for future investigations and the findings and conclusions have important implications for our understanding of the conservation of bilaterian body plans. I have only rather minor comments that should help to further improve the paper:

We thank the reviewer for the time taken to read our manuscript, and for the very positive feedback on our work. Below are our point-by-point comments.

1)      My major point is the writing. It is at many places convoluted and wordy. While the manuscript unfortunately does not contain any page or line numbers to point at specific positions, I counted nearly 19 pages of the Results part (after deducting the figures). While the discussion (here called Summary and conclusion) is shorter, it still could be more succinct. The current style is heavy on the reader and hence dilutes the interesting main messages. I strongly advise to drastically shorten the main manuscript and perhaps push part of the detailed result descriptions to the supplement.

We first and foremost want to sincerely apologise again to all reviewers for the absence of page and line numbers in our original submission, and for the difficulties this created. Page and line numbers have bee now added in the revised version (and we refer to these new page numbers in our responses below). We have also now more appropriately renamed the “Summary and conclusions” section as “Discussion”, as flagged by the reviewer.

Following feedback from all reviewers, we have now significantly shortened and simplified the entire manuscript, clarified key points of ambiguity, edited down tangential sections of text, as well as significantly re-structured the Discussion section, aiming to facilitate reading and better highlight the main messages of the paper.

2)      The discussion is relatively short and overall is touching on a number of interesting questions, e.g. the question of whether the larva or the adult is ancestral. Along this line, I think they also could discuss their findings in the context of a related longstanding question in EvoDevo, i.e. whether the urbilaterian was simple (acoel worm like) or complex. Likewise, I find it interesting that the conservation is more pronounced among those protostome and deuterostome species that have a primary ciliary larva, compared to the direct developers in the two chordates. The authors are invited to speculate about the underlying cause for this correlation.

We thank the reviewer for the precious feedback and for the invitation for further speculation. We however also note that other reviewers highlighted the Discussion as being maybe excessively speculative and given the overall highlighted need to streamline and shorten the manuscript, we feel like furthering lengthening the Discussion will not be overall welcomed. Still, we thank the reviewer for the positive feedback on our speculative sections and note that we have re-written the section dedicated to the ancestrality of larval forms, which is now more balanced and well-rounded, and which now includes additional consideration for the alternative hypothesis.

The reviewer is absolutely correct in noting that the high degree of similarity between adult guts also happens to correspond to the three species (protostomes and deuterostomes) with ciliated larval stages. Though some elements for this correspondence do come from our reference to the sea urchin pattern, and could therefore simply derive from a bias introduced by our use of an invertebrate reference, this higher degree of similarity remains clear even based on our newly-derived AP pattern (i.e. module markers), which was instead obtained from a cross-species approach that integrated all five species. Even then, we believe that any speculation on the evolutionary implications of such an observation would require more species, and most notably the transcriptomic analysis of adult guts from invertebrate species *without* ciliary larval intermediates (protostome and deuterostome). In summary, we do believe that such higher degree of similarity may have an evolutionary/biological grounding, possibly associating variation in larval intermediates with variation in through gut domains, but we do also believe that the still limited number of species we considered does not allow us to address this point appropriately.

Similar considerations apply to the configuration of the urbilaterian. Ultimately, and to the extent that we can speculate from our observed conservations of adult gut features, we would propose that the urbilaterian was in fact a fairly complex, through-gutted organism (i.e. not an acoel-like worm). Specifically, the urbilaterian ancestor would posses a through-gut with the structural and functional features summarised in the top panel of our Fig 7A. Still, the uncertainty of these inferences remains, and more specific statements would require, barred unlikely archaeological finds, the inclusion of guts from a much larger selection of extant species. Even more importantly, it would require a comprehensive and systematic survey of the module markers we identified, across the gut and other tissues of non through-gutted organisms (see e.g. Andrikou et al., 2025). If anything, we hope that our findings may spur renewed interest in the exploration of *adult* bilaterian guts, which in turn may find in our data and in our study a useful grounding. Of note, even our depiction of the urbilaterian silhouette in Fig 7C was just tentative, and we now fade it to further indicate its speculative nature. In fact, most recent archaeological analyses may suggest again higher support for a sessile ancestor with a J-shaped gut (Martynov & Korshunova, 2022).

We are overall very appreciative of the feedback and remain comforted to know that our paper is able to spark further interest for such speculations, which we are confident will greatly benefit the field of through-gut eco-evo-devo. Though our still limited roster of species may not allow more definitive answers at the moment, we do believe that our work will be able to act as an important anchor and justification for any such future investigations in these directions.

3)      Some TF expression patterns appear to be less conserved between sea urchin and the other species. The authors suggest that they may rather be terminal markers, instead of anterior or posteriormost markers. Q: are they conserved among the others and is the sea urchin the outlier? If yes, is that perhaps linked to the inversion of part of the hox cluster within the genome?

The genes we here tentatively define as “terminal” are genes that in some species are anterior markers, in other species are posterior markers, and in other more are anterior and posterior. At least from the genes we have investigated, we do not see the sea urchin pattern as necessarily an outlier. In response to other reviewers’ comments, we now make this clearer within the main text, and we also further refer this reviewer to the data in our answer to Reviewer 2’s comment about the *tbxt* expression pattern for another example of such potential terminal genes with poor conservation compared to sea urchin. Though we have not found a way to address this systematically, Sp does not appear to be an outlier with respect to the other four species, and each species generally shows alternative expression of such genes at either terminus. We do welcome more targeted investigations on the matter, which in fact would find in our dataset a very valid basis. In our already dense manuscript however, we could not justify such an ulteriorly focused investigation on the sea urchin only, and on the patterning consequences of its Hox cluster inversion.

Still, we want to highlight the fact that even though sea urchin shows a partial inversion of the Hox cluster within the genome, we do still find that these genes are expressed collinearly to the standard configuration: that is, anterior Hox genes are expressed in the anterior segments of the sea urchin through-gut, and not posterior to the posterior Hox genes, as would be expected if they were to follow the genomic arrangement. In turn, this would suggest that such a rearrangement does not appear to necessarily translate to the AP patterning of the sea urchin gut.

4)      Along the same line: Previous studies have shown by in situ hybridization that for instance FoxA2 and Bra (TbxT) are expressed in both proctodeum and stomodeum in sea urchins, sea stars and also Platynereis (e.g. Arendt et al., 2001). However, in the heatmap in Fig. 3A, these genes are detected only at one end. Is it possible that they have simply not be detected at the respective other end (or not as differentially expressed)? If yes, these genes (e.g. FoxD4, NeuroD) might be indeed terminal genes.

We first and foremost want to confirm that the genes and patterns plotted in Fig 3 are already downstream of relatively strict/conservative filtering thresholds (we refer the reviewer to our answer to the first comment by Reviewer 1, in which we detail this much more at length). In other words, our dataset is penalised by false negatives (i.e. filtered out genes with low expression, even though they might in fact still have a biological role within such low expression levels), and there is instead no risk that we missed higher expressed levels of expression reporting them as low. Certainly, the z-score based representation can amplify perceived expression differences between segments, and we have now addressed this by also including absolute counts data as an overlay of key figure panels (including Fig 3, see again our comments to Reviewer 1). We believe this change now allows readers to fully appreciate the effective expression of specific genes across gut segments.

If our interpretation is correct, we believe there has been a slight misunderstanding of our definition of “terminal genes”, which was indeed clearly ambiguous as flagged by Reviewer 2, and which we have accordingly strengthened in our revised manuscript. We did not want to imply that “terminal genes” are necessarily expressed at both termini in all species, but that they are so when considered and summarised at the pan-bilaterian level. Though our “terminal genes” do include genes that are expressed at both gut termini of a given species (such as *foxD4* in Platynereis, see Fig 3B, or such as *Drosophila* terminal genes, see our comments to Reviewer 2), genes that are only expressed at one terminus within a given species can also be classified as “terminal genes”. We classify such genes as “terminal genes” when we see that in other species they flip terminus. *FoxP4* is such a gene: anterior in Pd, Sp, Bf, and Ao, and posterior in Pf.

We however thank the reviewer for raising this point, and for bringing into the discussion what was observed in larval stages. As noted, we find that many of the genes we were finding “non-conserved” and flipping across adult gut termini, are also genes which are expressed at both ends of the larval stages of many species. With our comparison with the sea urchin larva, we show that some “anterior” or “posterior” markers are in fact expressed at both ends across ontogeny, first in one end in the larva, later in the other end as a juvenile. There is, in summary, a predisposition to flipping that we see for the same genes across developmental stages of the same species, across adult guts of different species, and across gut of different larvae. Rather than interpreting these flips as fundamental changes in evolution or in the regulatory identity of these termini, we instead speculate that these patterns may more simply reflect, in practice, a more general terminal role of these genes. Clearly, and given our absence of larval AP transcriptomic data, this is not something we can investigate systematically, and we therefore preferred to refrain from elaborating on this within the manuscript. We thank however the reviewer for highlighting key evidence from the larval expression patterns of key patterning genes, because they remain a key element to the full characterisation of the true EvoDevo function of these genes. To some extent, this is indeed what we wanted to point at with panel B of our summary Fig 7.

5)      To make the important point that many of the genes patterning the adult gut represent embryonic developmental regulators that are maintained or redeployed (after sometimes drastic metamorphosis), they rely on the published data of the sea urchin larval gut (Annunziata et al., 2014). Are similar data also available for Platynereis or P. flava larva? An early study on Platynereis larvae (Arendt et al., 2001) points in this direction, but more recent single cell data might be useful in the regard. This could potentially strengthen the authors conclusions on this point.

This is a critical point, and we thank the reviewer for it. To the best of our knowledge, and at the time of our analysis, no equivalent (published) dataset existed for the larval gut of any of the three other species we considered, and the data and work from Annunziata et al., 2014 remains a rare and extremely precious resource in its integrated linking of gene expression patterns (though still not at the transcriptome level) to precise anatomical domains. It is in fact only because of this fact that we initially used the sea urchin expression sequence as a reference for the other four species, noting however that even such sequence (Annunziata et al., 2014) was itself the integrated result of extensive expression data across larval guts, and applies beyond the sea urchin (Boyle et al., 2014, and indeed Arendt et al., 2001). If anything, therefore, we highlight the lack of transcriptome-wide, anteroposteriorly-ordered data for bilaterian larval guts (as well as juvenile and adult ones). We sincerely thank the reviewer for raising this point: single-cell level data of metamorphic guts of vertebrate and invertebrate species is still lacking, is strongly needed, and holds important value.

We do note that one such dataset, which we discuss in the text, was published for a close sea urchin species as we were finalising our comparative bulk transcriptomic analysis. This is the Paganos et al 2025 dataset for *Paracentrotus lividus*. As we are writing this comment, another of such datasets was just published for the hemicordate *Schizocardium californicum* (Bump et al., 2025: “Major cell type differences between larval and adult hemichordate body plans”). Crucially, both datasets integrate single-cell data (including, among other tissues, the gut) at both their larval and their juvenile configuration and could be used as a resource for gut-focused investigations.

For what concerns protostome species, critically, a single-nucleus atlas of the 6dpf *Platynereis dumerillii* larva has also just been published as we are writing this comment (Oel, Cros, Pan, Buglakova, Haury Parra et al., 2025: “A whole-body cell type atlas mapped into an electron microscopy volume of an annelid worm”). Though the study itself focuses on neuronal subtypes, it is crucial to note that data for nuclei with assigned midgut identity (*GATAe+, hnf4+*) as well as exocrine and endocrine pancreatic signatures is included, and likely to be soon made available.

We believe our data provides a solid guide to analogous investigations within such new datasets, especially since in many cases AP position can still be imputed even in single-cell datasets, despite dissociation. For species where such datasets are missing, we hope that our findings in adults and based on bulk transcriptomic can highlight the type of data that is still missing, and can support such data collection efforts.

6)      Are there genes shared in their AP pattern among at least 2 species that are not found in sea urchin? In other words, is the sea urchin a good reference?

Though we have not addressed this systematically, we can find combinations of markers that are conserved for any pair of species, and not sea urchin, regardless of phylogenetic position. We can also find species-specific sequences of anteroposterior markers that perfectly define anteroposterior position for a given species, and which will not hold for the sea urchin. To just mention a marker discussed at length in a previous comment (see Reviewer 2), the very *Pdx* expression pattern in adult sea urchin is highly anomalous compared to all other species and defines a posterior-high gradient in the sea urchin stomach. Other immediate examples may be the pattern of expression of *Hox6* and *Hox7*, which in the adult sea urchin gut define an expression domain around the stomach-intestine sphincter rather than showing graded expression patterns (Fig 2B).

As mentioned in a previous comment, we therefore want to clarify that we did not use the sea urchin as a reference (in the initial steps of our investigation) because we believe this species to be particularly representative of e.g. an ancestral through-gut configuration, but only because evolutionarily-grounded, gut-specific patterning data has its strongest literature support in the sea urchin, and specifically in the summary presented by Annunziata et al., 2014. Coincidentally, this is also one of the species included in our adult gut roster, which allowed us to directly elaborate on larval-to-adult correspondences.

Crucially, analogous investigations could be done taking any of the other four species as a starting point and then assessing conservation and divergence across the remaining organisms. Such investigations would have had much less grounding in existing literature, but indeed clearly highlight that our main conclusions about the AP organisation of adult guts could not be based on referencing to any single species.

This is precisely why we quickly move to an unbiased analysis considering all possible transcription factors shared across all five species. That is, regardless of the species taken into consideration, we only identify transcription factors that are consistently found at equivalent anteroposterior positions in all five species. In other words, these are TFs that would be found to be conserved across all species regardless of which species one would have taken as a reference.

**Cited work:**

**Kerstjens**, S., Engert, F., Zador, A. M., & Douglas, R. J. (2024). Eigengene reveals invariant global spatial patterns across mouse and fish brain development (p. 2024.09.19.613507). bioRxiv. <https://doi.org/10.1101/2024.09.19.613507>

**Paganos**, P., Ronchi, P., Carl, J., Mizzon, G., Martinez, P., Benvenuto, G., & Arnone, M. I. (2022). Integrating single cell transcriptomics and volume electron microscopy confirms the presence of pancreatic acinar-like cells in sea urchins. Frontiers in Cell and Developmental Biology, 10. <https://doi.org/10.3389/fcell.2022.991664>

**Paganos**, P., Ullrich-Lüter, J., Almazán, A., Voronov, D., Carl, J., Zakrzewski, A.-C., Zemann, B., Rusciano, M. L., Sancerni, T., Schauer, M., Akar, O., Caccavale, F., Cocurullo, M., Benvenuto, G., Croce, J. C., Lüter, C., & Arnone, M. I. (2025). Single Nucleus Profiling Highlights the All-Brain Echinoderm Nervous System (p. 2025.03.24.644250). bioRxiv. <https://doi.org/10.1101/2025.03.24.644250>

**Mantica**, F., Iñiguez, L. P., Marquez, Y., Permanyer, J., Torres-Mendez, A., Cruz, J., Franch-Marro, X., Tulenko, F., Burguera, D., Bertrand, S., Doyle, T., Nouzova, M., Currie, P. D., Noriega, F. G., Escriva, H., Arnone, M. I., Albertin, C. B., Wotton, K. R., Almudi, I., … Irimia, M. (2024). Evolution of tissue-specific expression of ancestral genes across vertebrates and insects. Nature Ecology & Evolution, 8(6), 1140–1153. <https://doi.org/10.1038/s41559-024-02398-5>

**Andrikou**, C., Pang, K., Børve, A., Lu, T. M., & Hejnol, A. (2025). Molecular evidence from xenacoelomorph gonopore formation supports homology with the bilaterian anus. Nature Ecology & Evolution, 1-11.

**Arendt**, Detlev, Ulrich Technau, and Joachim Wittbrodt. "Evolution of the bilaterian larval foregut." Nature 409, no. 6816 (2001): 81-85.

**Boyle**, M. J., Yamaguchi, E., & Seaver, E. C. (2014). Molecular conservation of metazoan gut formation: Evidence from expression of endomesoderm genes in Capitella teleta (Annelida). EvoDevo, 5(1), 39. <https://doi.org/10.1186/2041-9139-5-39>

**Bump**, Brewster, Formery, Lubeck, Campbell, Morri, Sit, Rokhsar, Benham-Pyle, Lowe (2025). “Major cell type differences between larval and adult hemichordate body plans”

bioRxiv 2025.10.31.685866; doi: <https://doi.org/10.1101/2025.10.31.685866>

**Oel**, **Cros**, **Pan**, **Buglakova**, **Haury Parra**, Santangeli, Gerber, Papalopoulos, Vergara, Puga, Disela, Niederhaus, Bertucci, Witte, Bilgesu, Quintana, Acevedo, Musser, Kreshuk, Arendt (2025). “A whole-body cell type atlas mapped into an electron microscopy volume of an annelid worm” bioRxiv 2025.10.30.685666; doi: <https://doi.org/10.1101/2025.10.30.685666>
